# Pathological TDP-43 changes in Betz cells differ from those in bulbar and spinal *α*-motoneurons in sporadic amyotrophic lateral sclerosis

**DOI:** 10.1007/s00401-016-1633-2

**Published:** 2016-10-18

**Authors:** Heiko Braak, Albert C. Ludolph, Manuela Neumann, John Ravits, Kelly Del Tredici

**Affiliations:** 1 Clinical Neuroanatomy Section, Department of Neurology, Center for Biomedical Research, University of Ulm, Helmholtzstrasse 8/1, 89081 Ulm, Germany; 2 Department of Neurology, University of Ulm, Ulm, Germany; 3 Department of Neuropathology, University of Tübingen, Tübingen, Germany; 4 German Center for Neurodegenerative Diseases (DZNE), Tübingen, Germany; 5 Department of Neurosciences, ALS Translational Research, University of California (San Diego), La Jolla, USA

**Keywords:** *α*-Motoneurons, Amyotrophic lateral sclerosis, Betz cells, Motor neuron disease, Primary motor cortex, TDP-43, TAR DNA-binding protein, Therapeutics, Transsynaptic spreading

## Abstract

Two nerve cells types, Betz cells in layer Vb of the primary motor neocortex and *α*-motoneurons of the lower brainstem and spinal cord, become involved at the beginning of the pathological cascade underlying sporadic amyotrophic lateral sclerosis (sALS). In both neuronal types, the cell nuclei forfeit their normal (non-phosphorylated) expression of the 43-kDa transactive response DNA-binding protein (TDP-43). Here, we present initial evidence that in *α*-motoneurons the loss of normal nuclear TDP-43 expression is followed by the formation of phosphorylated TDP-43 aggregates (pTDP-43) within the cytoplasm, whereas in Betz cells, by contrast, the loss of normal nuclear TDP-43 expression remains mostly unaccompanied by the development of cytoplasmic aggregations. We discuss some implications of this phenomenon of nuclear clearing in the absence of cytoplasmic inclusions, namely, abnormal but soluble (and, thus, probably toxic) cytoplasmic TDP-43 could enter the axoplasm of Betz cells, and following its transmission to the corresponding *α*-motoneurons in the lower brainstem and spinal cord, possibly contribute in recipient neurons to the dysregulation of the normal nuclear protein. Because the cellular mechanisms that possibly inhibit the aggregation of TDP-43 in the cytoplasm of involved Betz cells are unknown, insight into such mechanisms could disclose a pathway by which the development of aggregates in this cell population could be accelerated, thereby opening an avenue for a causally based therapy.

## Introduction

The pathology of sporadic amyotrophic lateral sclerosis (sALS) is associated with the dysregulation of the 43-kDa transactive response DNA-binding protein (TDP-43) [3, 4, 37, 49, 54, 66] that leads to the formation of proteinaceous cytoplasmic aggregates in specific cortical and subcortical projection neurons with long axons, whereas cells with short axons are spared [27, 60]. The Betz giant pyramidal cells in layer Vb of the primary neocortical motor cortex [14] and the *α*-motoneurons of the lower brainstem and spinal cord belong to the cell types that develop TDP-43 pathology early in the disease process [13].

In healthy nerve cells, TDP-43 is a predominantly intranuclear protein and its expression can be visualized using immunohistochemistry. However, the protein also can migrate from the nucleus into the cytoplasm of the somatodendritic and axonal compartments and then re-enter the nucleus [4, 28, 41, 54, 64]. Furthermore, the factors initiating the dysregulation of intranuclear TDP-43 are not known. Use of antibodies directed against the native protein shows that in neuronal types susceptible to sALS a reduction or absence of normal nuclear TDP-43 immunoreactivity is associated with TDP-43 mislocalization and inclusion body formation [28, 54, 66]. During its cytoplasmic phase, the protein becomes abnormally phosphorylated (pTDP-43) and possibly undergoes a conformational change [34, 41, 55, 69]. In its abnormal and ubiquitinated state, the protein can no longer re-enter the cell nucleus, nor can it be metabolized or eliminated by autophagy, proteosomal recycling, or other endogenous cellular removal mechanisms [31, 41, 54, 60].

In sALS, the cell nucleus eventually forfeits its TDP-43 immunoreactivity, and the cell soma, dendrites, and axon of most involved neurons gradually develop skein- or dash-like aggregates consisting of abnormally phosphorylated, ubiquitinated, and aggregated pTDP-43 [10, 32, 52, 55, 57]. Antibodies that only recognize pTDP-43 do not show the protein’s displacement from the nucleus into the cytoplasm; however, they do visualize the cytoplasmic aggregates and facilitate their recognition [32, 55].

The pTDP-43 pathology appears to progress in a sequential manner and, for this reason, shifts in the regional distribution of the lesions have been used to propose the differentiation of four neuropathological sALS stages [13]. More recently, the same pattern of pathological changes has been reproduced in neuroimaging-based studies [33, 36, 53, 67]. In stage 1, pTDP-43 pathology is found in the Betz cells of Brodmann field 4 [38] in the neocortex and in bulbar and spinal *α*-motoneurons with the exception of the motoneurons that control the extrinsic eye muscles [1, 13]. The topographical distribution of the lesions in stage 2 is marked by the development of pTDP-43 inclusions in parvocellular projection neurons of the red nucleus and, in stage 3, in medium-sized projection neurons of the caudate nucleus and putamen [13]. During stage 4, pTDP-43 lesions develop in allo- and neocortical regions of the cerebral cortex [12, 13, 23, 36].

In the present study, based on 15 cases staged for pTDP-43 pathology, we report the finding that pathologically altered TDP-43 in Betz cells reacts differently than that in bulbar or spinal *α*-motoneurons. The major differences between the two types of histological profiles are discussed within the context of their possible consequences and implications for the potential further progression or spread of the pTDP-43 lesions.

## Materials and methods

### Study cohort

Fifteen (*N* = 15) cases with a clinically and neuropathologically confirmed diagnosis of sALS [16, 47, 48] staged according to a recently published protocol [13] and three controls were included. This retrospective study was performed in compliance with university ethics committee guidelines as well as German federal and state law governing human tissue usage. Informed written permission was obtained from all patients and/or their next of kin. Demographic and clinical data of sALS cases (4 females, 11 males, 36–76 years of age) and controls are summarized in Table 1.

**Table 1:** Data from the sporadic ALS cases (*N* = 15) and controls (*N* = 3) studied

ALS cases	Age	F/M	Dis onset	Dis dur	ALS	NFT	Aβ	PD	*α*-Motoneurons bulbar/spinal
1	55	M	Arm	2.3	1	I	0	0	(N−, C+)**/**(N−, C+)
2	61	M	Arm	2.0	1	I	0	3	(N−, C+)**/**(N−, C+)
3	74	M	Respiratory and trunk	1.75	1	I	0	0	na**/**(N−, C+)
4	51	F	Arm	2.11	1	I	0	0	(N−, C+)**/**(N−, C+)
5	61	M	Leg	2.0	2	I	1	0	(N−, C+)**/**(N−, C+)
6	53	M	Arm	0.9	2	I	0	0	(N−, C+)**/**(N−, C+)
7	58	M	Arm	2.5	2	I	0	0	(N−, C+)**/**(N−, C+)
8	49	F	Arm	3.5	2	I	0	0	(N−, C+)**/**na
9	36	M	Bulbar	3.0	2	I	0	0	(N−, C+)**/**(N−, C+)
10	68	M	Arm	2.0	3	I	0	0	(N−, C+)**/**(N−, C+)
11	57	M	Leg	2.0	4	III	0	0	(N−, C+)**/**(N−, C+)
12	64	F	Arm	1.3	4	II	0	0	(N−, C+)**/**na
13	68	M	Leg	3.6	4	I	0	0	(N−, C+)**/**(N−, C+)
14	76	F	Bulbar	1.4	4	II	0	0	(N−, C+)**/**(N−, C+)
15	57	M	Arm	2.1	4	I	0	0	(N−, C+)**/**(N−, C+)
Controls									
16	57	M	na	0	0	I	0	0	na
17	54	F	na	0	0	II	1	0	na
18	73	M	na	0	0	I	1	0	na

*M/F* male, female; *Age* age at death (in years); *Dis onset* region of initial clinical disease signs; *Dis dur* disease duration (in years); *ALS* neuropathological stages 1–4 of sporadic amyotrophic lateral sclerosis; *NFT* Alzheimer-related neurofibrillary tangles stages I–VI (Gallyas silver-iodide staining); *Aβ* Amyloid-β deposition phases 1–4; *PD* Parkinson’s disease-related neuropathological stages 1–6 (*α*-synuclein immunohistochemistry); *bulbar/spinal α*-motoneurons in the medulla oblongata (hypoglossal nucleus, facial nucleus) and *α*-motoneurons of the spinal cord anterior horn evaluated using lipofuscin pigment-staining combined with TDP-43 immunohistochemistry: (*N* ***−***) α-motoneurons with a TDP-43 immunonegative cell nucleus, (*C+*) presence of TDP-43 immunoreactive cytoplasmic lesions, na not available (in controls = not applicable)

## Tissue embedding, sectioning, and staining

Brainstems, a single hemisphere, and tissue blocks from the spinal cord were fixed by immersion in a 4 % buffered aqueous solution of formaldehyde for 10–14 days (Table 1, cases #7, 10–18) or for a minimum of 90 days (Table 1, cases #1–4, 6, 8, 9). After fixation, the hemispheres were cut perpendicular to Forel’s axis using a macrotome into 1 cm thick slices and embedded in polyethylene glycol (PEG 1000, Merck) according to a previously published protocol [11]. Tissue sectioning was performed with a tetrander (Jung, Heidelberg, Germany) at a thickness of 100 µm to insure that the Betz cells could be studied in their entirety without sectioning artifacts, i.e., at a thickness exceeding the average diameter of a giant pyramidal Betz cell [5, 38].

**Fig. 1:**
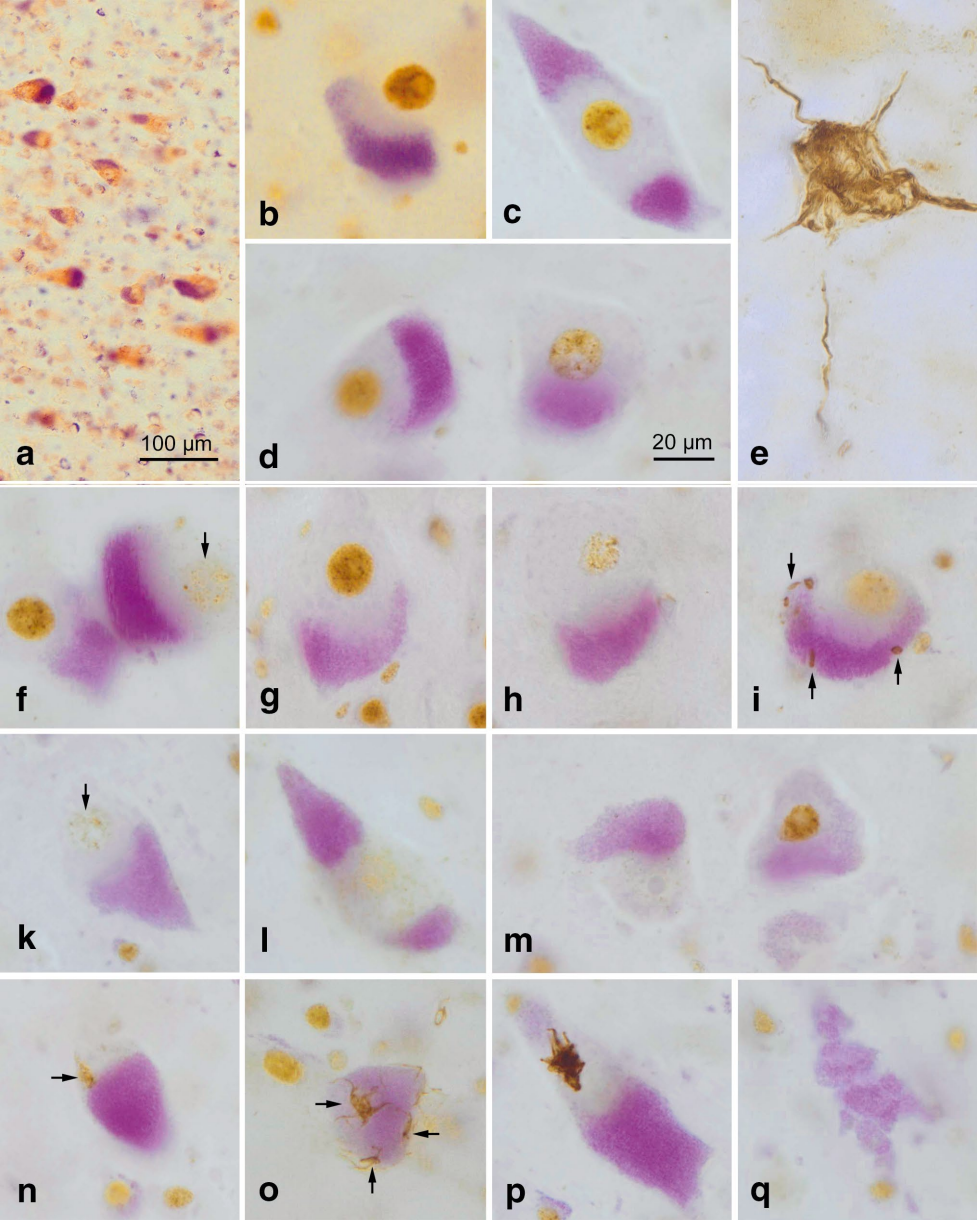
**a** Overview of the Betz giant pyramidal cells in layer Vb of the primary motor neocortex of a control individual (Table 1, case #17). Layer V is shown at right angles to the cortical surface (*at left*). The pigment-Nissl staining (*violet*-*red*) here and the pigment-staining (*violet*) in the remaining micrographs marks the location of the closely packed lipofuscin pigment granules within the cell somata. **b**–**d**, **f**–**q** Pigment-staining and TDP-43 immunohistochemistry in individuals with sALS. **b**, **f** (*at left*), and **g** Micrographs showing normal Betz cells with strong nuclear TDP-43 immunostaining. **c**, **d** A Betz cell with reduced nuclear TDP-43 immunostaining (**d**, *at left*) alongside of Betz cells in which TDP-43 immunostaining is nearly absent (**d**, *at right*; see also **h**, **i**) or absent (**f**, *at right*, **k** and **l**, *at left*; see also **m**, **n**, **o**). **e** A pTDP-43-immunopositive *α*-motoneuron filled with aggregates in the motor nucleus of the hypoglossal nerve (N. *XII*) (Table 1, case #13). In the other micrographs here, the Betz cells that become involved in sALS do not display this kind of lesional profile. **h**–**m** These micrographs show the growing reduction and loss of intranuclear TDP-43 immunoreactivity in Betz cells; note also, however, that the cytoplasmic inclusions (aggregates) that do develop are slight. **i** A Betz cell containing discreet dot-like or granular cytoplasmic inclusions (*arrows*). An exception to this discreet cytoplasmic pathology is seen in **p**. **m** A Betz cell with a completely ‘empty’ (i.e., TDP-43 immunonegative) cell nucleus (*at left*) shown next to a Betz cell with normal nuclear TDP-43 immunostaining. In the cell at the left, only the nucleolus is still visible. **n**, **o** Subtle TDP inclusions (*arrows*) in Betz cells, including skein-like lesions in **o**. **q** Remnants of lipofuscin pigment granules mark the site of a dead Betz cell. *Scale bar* in **d** is valid for all micrographs except **a**. 100 µm polyethylene glycol (PEG) sections. Micrographs **b**, **o** (Table 1, case #1), **c**, **d**, **f**, **k**–**n**, **p**, **q** (Table 1, case #3), **g**–**l** (Table 1, case #2). A plan apochromat 40:1 lens was utilized to evaluate and photograph individual Betz cells (**a**–**d**, **f**–**q**)

**Fig. 2:**
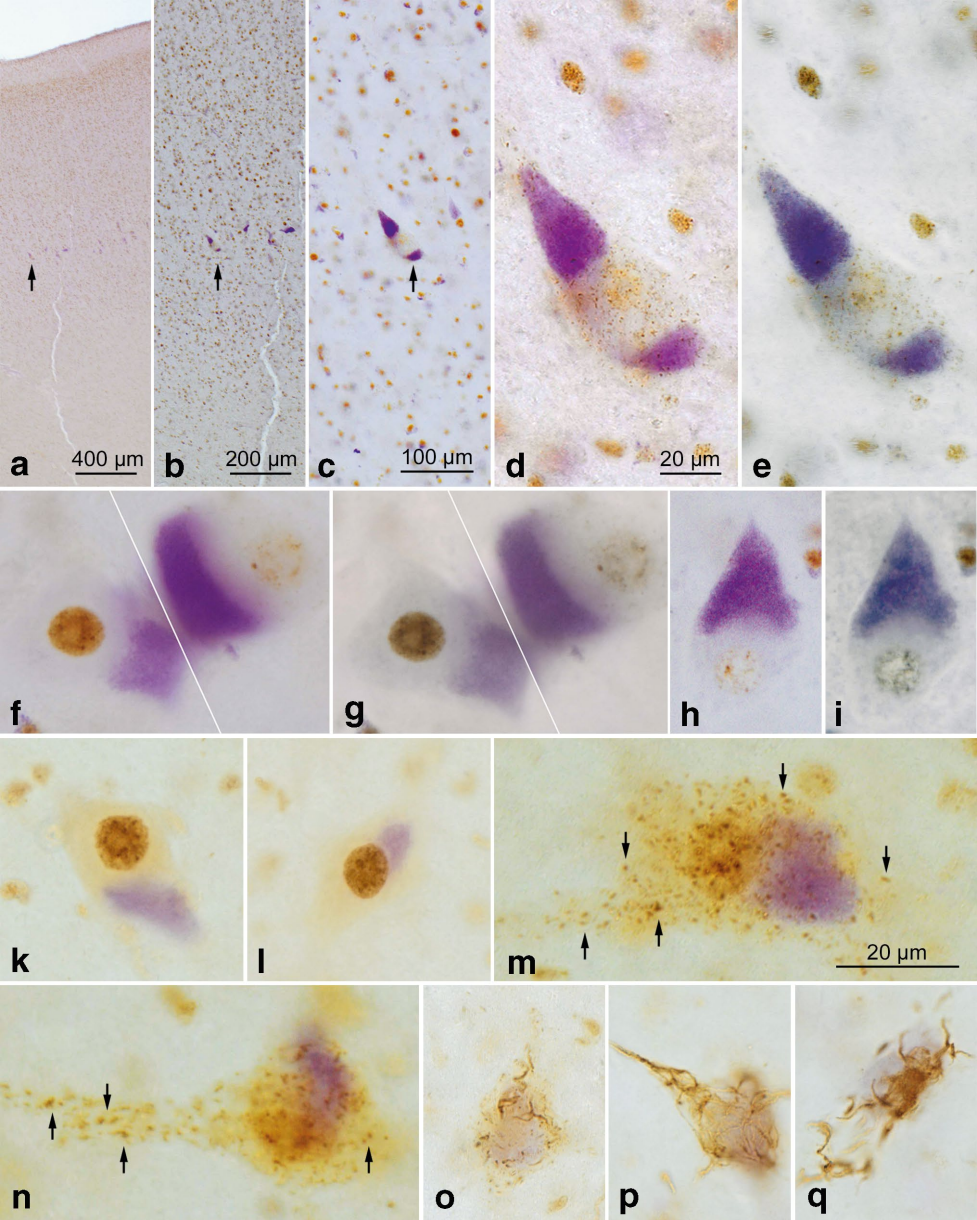
*Upper panels* **a**–**i** Betz cells as seen in sections stained for lipofuscin deposits (aldehyde fuchsin) combined with an immunoreaction for normal TDP-43 (chromogen: *brown*). Following an overview micrograph (**a**) and additional micrographs taken at higher magnifications (**b** and **c**), additional micrographs of individual Betz cells were taken using an apochromat 40:1 lens to show both an immunonegative cell nucleus and immunonegative cytoplasm of the cell body (**d**). Then, the coverslip was removed in xylene and the tissue section was transferred through a descending ethanol series to H_2_O before performing a second immunoreaction, this time using an antibody against phosphorylated TDP-43 (chromogen: *blue*). With the aid of the initial micrographs, the same cells of interest photographed previously were located in the same section again and studied in the double immunoreactions (**e**). Immunonegative cytoplasm of the cell body in immunoreactions for normal TDP-43 remained immunonegative after the second immunoreaction for phosphorylated TDP-43 was performed, i.e., no inclusion bodies or traces of aggregated material were found that only appeared in immunoreactions with phosphorylated TDP-43 but remained immunonegative in sections with normal TDP-43 immunostaining. **f** and **g**, **h** and **i** Additional pairs of cells were visualized in the manner described above. **f**, **g** Here, an involved Betz cell with an empty cell nucleus and empty cytoplasm is located directly adjacent to a normal Betz cell displaying a strongly immunopositive cell nucleus. Micrographs **a**–**i** (Table 1, case #3). *Lower panels*, **k**–**q** Bulbar *α*-motoneurons of the hypoglossal nerve. **k** and **l** Staining of lipofuscin deposits (aldehyde fuchsin) combined with an immunoreaction for normal TDP-43 (chromogen: *brown*) sometimes revealed normal *α*-motoneurons with strongly immunopositive cell nuclei. **m**–**n** With the onset of nuclear clearing, i.e., when a weakly immunoreactive cell nucleus clearly was discernible, the formation of cytoplasmic dash-like TDP-43-immunopositive particles (*arrows*) began to develop in the cell soma, and these particles were widely distributed there. **o**–**q** A possibly more advanced phase in the development of the TDP-43 lesions could be the coalescence of the dash-like particles into the fine (**o**) and coarser (**p** and **q**) forms of skein-like inclusion bodies. In contrast to the situation encountered in the Betz cells, *α*-motoneurons with immunonegative cell nuclei that were accompanied by an immunonegative cytoplasm did not occur. *Scale bar* in **d** is valid for **e**–**l** and **o**–**q**. 100 µm polyethylene glycol (PEG) sections. Micrographs **k**–**n** (Table 1, case #2), **o** (Table 1, case #1), **p**, **q** (Table 1, case #13). A plan apochromat 40:1 lens was utilized to evaluate and photograph individual Betz cells (**a**–**i**)

One set of free-floating sections from each case underwent silver-staining with the Gallyas silver-iodide method, as described previously, to visualize argyrophilic neurofibrillary lesions associated with Alzheimer’s disease [11]. For topographical orientation, a second set of free-floating sections from all tissue blocks for each case was pretreated with performic acid and processed with aldehyde fuchsin for selective staining of lipofuscin deposits combined with a basophilic Nissl stain (Darrow red). The pigment-Nissl staining technique makes it possible to differentiate reliably among different neuronal types based on their lipofuscin profiles and also to assess neuronal loss owing to the presence of pigment remnants lying free in the neuropil (Fig. 1q) [6–8, 11]. Sections from Brodmann field 4 were chosen that showed the midline region (interhemispheric fissure) of the primary motor neocortex because the Betz cells attain their maximal dimensions there, where their voluminous somata and large uniformly roundish nuclei make them easy to recognize (e.g., Figs. 1b, c, f, g, 2a–i). In adults, these cells contain very large and smoothly contoured deposits of densely packed lipofuscin granules, usually located in basal portions of the soma. The pigment-Nissl technique shows all of these distinctive features of human Betz cells [6, 7].

## Immunohistochemistry

pTDP-43 pathology was analyzed in a third set of free-floating 100 µm hemisphere sections following performic acid pretreatment and staining with aldehyde fuchsin using a commercially available ps409/410-TDP rabbit polyclonal antibody (1:10000; Cosmo Bio, Carlsbad, CA, USA). Following performic acid pretreatment and staining with aldehyde fuchsin immunohistochemistry was performed on a fourth set of free-floating sections with a rabbit polyclonal antibody recognizing N-terminal TDP [1:5000; Proteintech, Manchester, UK] to visualize normal nuclear TDP-43 [70]. Selected pigment-Nissl stained 100 µm sections containing the spinal cord anterior horn and the motor nucleus of the hypoglossal nerve (N. XII) were also immunostained for TDP-43, and double immunostaining (pTDP-43 plus TDP-43) was performed in four cases (Table 1, #1–4) on free-floating 100 µm sections that included the primary motor field. In these sections, pTDP-43 immunostaining was visualized using the SK-4700 blue chromogen (SG Substrate Kit, Vector, Burlingame, CA, USA) and TDP-43 staining was visualized with the brown chromogen 3,3′-diaminobenzidine tetrahydrochloride (DAB). Finally, three sets of free-floating sections were immunostained using the following antibodies: (1) a monoclonal antibody PHF-Tau (1:2000; Clone AT8; Pierce Biotechnology, Rockford, IL, USA [Thermo Scientific]) for hyperphosphorylated tau protein in pretangle material and neurofibrillary changes of the Alzheimer type [11]; (2) a monoclonal antibody anti-beta-amyloid (1:5000; Clone 4G8; Covance, Dedham, MA, USA) for detection of amyloid-*β* deposition [11, 71]; (3) a monoclonal mouse antibody anti-syn-1 (1:2000; Clone number 42; BD Biosciences, Mountain View, CA, USA) as a marker of Parkinson’s-related *α*-synuclein inclusions [9].

Tissue sections for immunohistochemistry were treated for 30 min in a mixture of 10 % methanol plus 10 % concentrated (30 %) H_2_O_2_ and 80 % Tris. Following pretreatment with 100 % formic acid for 3 min to facilitate the immunoreactions, blocking with bovine serum albumin was performed to inhibit endogenous peroxidase and to prevent nonspecific binding. Subsequently, each of the sets of free-floating 100 µm sections was incubated for 18 h at 20 °C using the primary antibodies. Subsequent to processing with a corresponding secondary biotinylated antibody (anti-mouse IgG, 1:200; Linaris) for 1.5 h, all immunoreactions were visualized with the avidin–biotin complex (ABC, Vectastain, Vector Laboratories, Burlingame, CA, USA) for 2 h and the chromogen 3,3′-diaminobenzidine tetrahydrochloride (DAB, D5637 Sigma, Taufkirchen, Germany). Omission of the primary antiserum resulted in non-staining. Positive as well as negative control sections were included. The tissue sections were cleared, mounted, and cover-slipped in a medium with a refraction index of 1.58 (Histomount, Thermo Fischer Scientific, Braunschweig, Germany, plus 10 % *α*-methylcinnamaldehyde). All sections were viewed and neuropathological staging was performed with an Olympus BX61 microscope (Olympus Optical, Tokyo, Japan). A plan apochromat 40:1 lens was utilized to evaluate and photograph individual Betz cells. Digital micrographs were taken with an Olympus XC50 camera using the analysis^®^ Soft Imaging System (Münster, Germany).

## Betz cells counts

To investigate what proportion of Betz cells in a single section displayed (A) a normal nuclear TDP-43 or (B) a reduced nuclear TDP-43 immunoreactivity in comparison to the proportion of those (C) showing complete loss of nuclear TDP-43 immunoreactivity with an empty somatic cytoplasm or with traces of aggregates, and to determine (D) how many Betz cell had been lost (the presence of lipofuscin pigment remnants in the immediately surrounding neuropil was used as a marker for cell loss), one observer (HB) examined a single slide from two sALS cases at neuropathological stage 1 (Table 1, #2, 3), two cases at neuropathological stage 4 (Table 1, #13, 14), each with a different site of clinical disease onset, and two controls (Table 1, #16, 18). In this manner, it was possible to arrive at approximate percentages for each of the four categories above (Table 2, A–D). Counting was performed on an Olympus BX61 microscope (Olympus Optical, Tokyo, Japan) at 200× magnification.

**Table 2:** Betz cell counts in *N* = 4 sporadic ALS cases and in *N* = 2 controls

ALS cases	Age	F/M	Dis onset	Dis dur	ALS	A	B	C	D
2	61	M	Arm	2.0	1	Total = 198**/**161 = **81** **%**	12 = **6** **%**	15 = **8** **%**	10 = **5** **%**
3	74	M	Respiratory and trunk	1.75	1	Total = 221**/**179 = **81** **%**	14 = **6** **%**	20 = **9** **%**	8 = **4** **%**
13	68	M	Leg	3.6	4	Total = 150**/**75 = **50** **%**	13 = **8** **%**	31 = **21** **%**	31 = **21** **%**
14	76	F	Bulbar	1.4	4	Total = 215**/**132 = **61** **%**	26 = **12** **%**	38 = **18** **%**	19 = **9** **%**
16	57	M	na	0	0	Total = 227**/**227 = **100** **%**	0	0	0
18	73	M	na	0	0	Total = 278**/**269 = **96** **%**	8 = **3** **%**	0	3 = **1** **%**

*M/F* male, female; *Age* age at death (in years); *Dis onset* region of initial clinical disease signs; *Dis dur* disease duration (in years); *ALS* neuropathological stages 1–4 of sporadic amyotrophic lateral sclerosis; *A* Total number of Betz cells in a single section and the percentage of those with a normal cell nucleus; *B* Betz cells with reduced nuclear TDP-43 immunoreactivity; *C* Betz cells displaying complete nuclear TDP-43 loss with an empty somatic cytoplasm or with traces of aggregates; *D* Betz cell loss based on the presence of lipofuscin pigment remnants in the neuropil

## Results

**Fig. 3:**
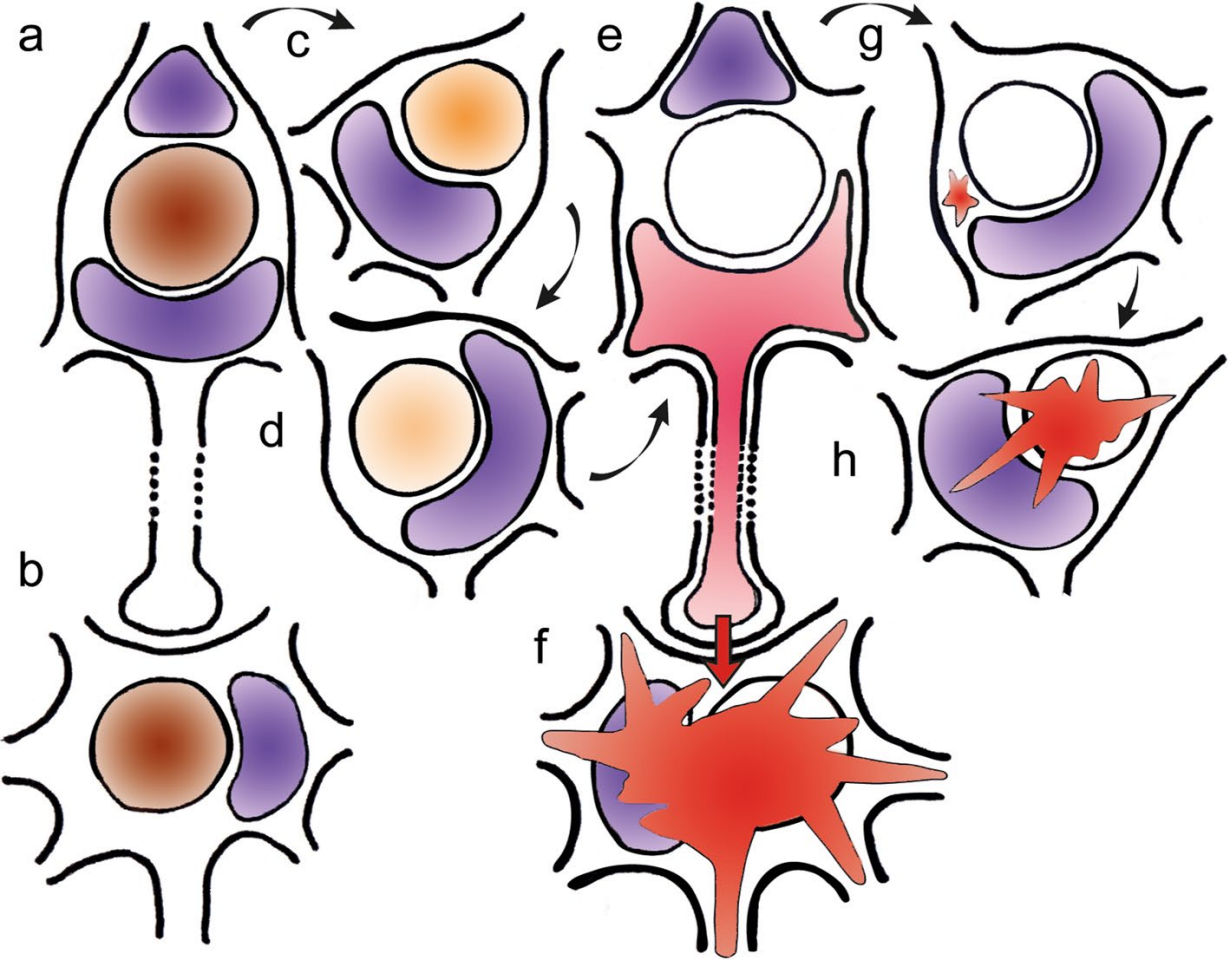
Schematic diagram summarizing the findings in giant Betz cells (**a**, **c**–**e**, **g**, **h**) and in *α*-motoneurons (**b**, **f**) in the cases of sporadic amyotrophic lateral sclerosis studied. We hypothesize that the pathology may be transferred by means of still soluble but toxic or pathogenic axoplasmic TDP-43 directly (monosynaptically) from involved Betz cells to *α*-motoneurons. This postulated route is marked by means of the *red arrow* in the synapse between **e** and **f**. Deposits of lipofuscin granules serve as a marker of the cortical cellular type and are represented here by *violet* shading. **a**, **b** Betz cells in controls as well as in non-involved Betz cells and in non-involved *α*-motoneurons displayed normal, strongly immunoreactive intranuclear TDP-43 staining (here, in *brown*). The long axon of the Betz cell projects to, and synapses directly on, the corresponding *α*-motoneuron in the lower brainstem or in the ventral horn of the spinal cord. **c**, **d** Involved Betz cells of the cases examined displayed increasingly weak (**c**) and severe reduction (**d**) of TDP-43 intranuclear immunostaining. **e** The putative end-point of this development may be reached when the cell nucleus is completely ‘empty’, i.e., TDP-43 immunonegative. Theoretically, the protein could be present (although no longer immunoreactive) in the somatodendritic and/or axonal cytoplasm in a soluble state (here, in *pink*), where, for an unknown time interval, it does not convert into insoluble aggregates. **f** At the same time the above-described abnormalities became visible within the Betz cells (depicted in **c**–**e**), somatic cytoplasmatic pTDP-43 inclusions were found in *α*-motoneurons (here, as *red* blasts). It should be emphasized that the aggregates were only found in the somatic and axonal cytoplasm. **g** Adjacent to the Betz cells that have TDP-43 immunonegative nuclei but lack pTDP-43 cytoplasmic inclusions, one sometimes encountered Betz cells containing discreet (dot-like, granular) aggregates (here, in *red*), which could indicate that these cells do not completely forfeit the potential to develop inclusion bodies. **h** Betz cells containing large aggregates were seldom. The *dashed lines* in **a** and **e** are intended to indicate that the involved axons are much longer than depicted schematically here

Beginning in neuropathological stage 1 of sALS and continuing thereafter in subsequent stages, the TDP-43 lesions in Betz cells differed from those in bulbar and spinal α-motoneurons in the following respects: (1) In Betz pyramidal cells, a TDP-43 immunonegative cell nucleus (Fig. 1m [at left]) or a weakly TDP-43-immunoreactive cell nucleus was marked by the absence of cytoplasmic inclusions (Figs. 1d [at right], f [at right], h, k, l, 2a–i) or by the presence of very mild cytoplasmic aggregates (Fig. 1i, n, o [arrows]). (2) By contrast, in involved bulbar and/or spinal *α*-motoneurons, a TDP-43 immunonegative cell nucleus was consistently accompanied by pTDP-43-immunopositive inclusions in the cytoplasm of the cell body (Table 1) (Figs. 1e, 2m–q; see also Fig. 3f diagram). All 15 sALS cases, including one individual with concurrent Lewy pathology (Table 1, case #2), displayed the staining profiles described in (1) and (2). Two of the controls (Table 1, cases #16, 17) were unremarkable. In the third control case (Table 1, #18), reduced TDP-43 nuclear staining and discrete cell loss were detectable in a small number of Betz cells (Table 2, #18 B, D).

In midline sections through the primary motor neocortex of sALS brains, not only a large number of uninvolved Betz giant pyramidal cells were observed in layer Vb between stages 1 and 4 but also noticeable fluctuations in the degree of their intranuclear TDP-43 immunoreactivity (Table 2). Betz cells in controls and uninvolved Betz cells of sALS cases displayed a vividly immunoreactive large cell nucleus. In addition to these normal and strongly immunostained cell nuclei (Figs. 1b, f [at left], g, 2f–g [at left]; see also Fig. 3a diagram), others in one and the same individual displayed reduced (Figs. 1c, d [at right], 3c) or remarkably weak intranuclear TDP-43 immunoreactivity (Figs. 1h, 3d). Indeed, in some instances, the intranuclear TDP-43 immunoreactivity was nearly absent (Fig. 1f [at right], k, l) or lacking altogether (Figs. 1m [at left], 2d, f [at right], h; see also Fig. 3e).

Notably, most Betz cells in which the intranuclear immunostaining was either extremely weak or absent also failed to display TDP-43-immunopositive cytoplasmic inclusions (Table 2; Figs. 1k, l, m [at left], 2d, f [at right], h; schematically depicted in Fig. 3c–e). This phenomenon was also present in the Betz cells of sections that underwent double immunostaining for TDP-43 and pTDP43, in which immunonegative cell nuclei were accompanied by immunonegative somata (shown by TDP-43 immunostaining and DAB, Fig. 2a–d, f [at right], h). The same cells then underwent a second immunoreaction, this time with pTDP-43 visualized by the SK-4700 blue chromogen. The somata did not display pTDP-43 immunoreactivity (Fig. 2e, g [at right], i). Only after repeated scrutiny of numerous Betz cells was it possible to find a few that contained slight traces of granular or even skein-like cytoplasmic pathology in their somata (Fig. 1i, n, o [arrows]; see also Fig. 3g). When present, these cytoplasmic aggregates were immunoreactive for both TDP-43 and pTDP-43, but none were solely pTDP-43-immunopositive [61]. It is unclear how long the absence of TDP-43-immunoreactive aggregates within the Betz cell somata persists. Alongside the Betz cells that lacked TDP-43 lesions and those bearing mild TDP-43 inclusions, some were eventually seen that contained much bulkier sALS-associated cytoplasmatic inclusions (Fig. 1p; see also Fig. 3h), and in some instances these inclusions attained the same size as the originally TDP-43 immunoreactive nuclei.

The abnormal changes observed in the Betz cells were accompanied as of stage 1 by pTDP-43 pathology in bulbar and/or spinal α-motoneurons (Table 1). The involved motoneurons displayed not only a loss of intranuclear TDP-43 immunoreactivity but, in contrast to the Betz cells, they showed the presence of pTDP-43 aggregates in their cytoplasm (Table 1; Figs. 1e, 2m–q; see also Fig. 3f). As in Betz cells, the shift within *α*-motoneurons of the hypoglossal nucleus began with gradually diminished intranuclear TDP-43 immunoreactivity (Fig. 2m, n). At that point––that is, when immunoreactivity of the cell nucleus was nearly absent––dash-like particles immunoreactive for both TDP-43 and pTDP-43 were found distributed uniformly within the somatodendritic compartment (Fig. 2m, n [arrows]).

## Discussion

Owing to their morphological profiles, human Betz cells are unique among other neurons of the neocortex [38]. Immunoreactions directed against all species of the TDP-43 protein (non-phosphorylated, phosphorylated, monomeric, and polymeric) were used to visualize the sALS-associated lesions in Betz cells and we supplemented the TDP-43 immunohistochemistry with a reliable staining technique for lipofuscin pigment granules (e.g., Figs. 1b, p, 2d–i) [6, 7]. Because the sALS-associated intranuclear changes are easily missed amidst the large number of normally immunostained Betz cell nuclei in layer Vb, the identity of the altered Betz cells with mislocalized (i.e., extranuclear) TDP-43 was facilitated using tissue sections near the midline of the primary motor neocortex at a thickness of 100 µm, which clearly exceeds the average Betz cell diameter of 50–60 µm [38]. Using the micrometer screw at all focal planes of the section, the cells in question were visualized in their entirety while minimizing the risk of assessing partially cut Betz cells, which would skew the results.

Double-immunoreactions directed against both TDP-43 and pTDP-43 of four of the cases studied showed that, in Betz cells with an immunonegative cell nucleus and a TDP-43-immunonegative soma, the cytoplasm was pTDP-43 immunonegative (Fig. 2a–i). The abnormalities seen in Betz cells (i.e., an immunonegative nucleus in the presence of an immunonegative cell soma) were not observable in sections containing bulbar or spinal *α*-motoneurons of the same cases. Instead, during the ‘emptying’ phase of their cell nuclei, involved *α*-motoneurons consistently showed the development of cytoplasmic inclusions, initially and usually in the form of fine dash-like particles (Fig. 2m, n) [10]. Some of the motoneurons displayed what could represent a possible further phase in the development of aggregates: namely, the coalescence of the fine dash-like particles into coarser but interconnected structures, which might have ended up as skein-like inclusions (Figs. 1e, 2o–q; see also schematic diagram in Fig. 3f). Motoneurons with an immunonegative cell nucleus and with an immunonegative cytoplasm were not seen.

Provided autopsy tissue is optimally fixed postmortem, immunohistochemistry performed on archival specimens is remarkably reliable [61]. Here, it is unlikely that the TDP-43 findings reported are attributable to staining variability or staining quality related to fixation times because in the vicinity of involved Betz cells with immunonegative nuclei we could observe in one and the same section uninvolved Betz cells that displayed immunopositive intranuclear staining. The quality of the pTDP-43 immunoreactions was also verified by comparing staining results in different brain regions of each sALS case studied. Insofar as all tissue samples from the same individual were subjected to identical fixation conditions, pTDP-43 staining artifacts can be excluded. The reduced TDP-43 nuclear staining and discrete loss of Betz cells in the oldest of the three controls (Table 2, case #18 B, D) is difficult to interpret. It might reflect an age-related change in the expression level of TDP-43 or an age-related altered nucleocytoplasmic transport machinery of postmitotic cells [19, 51].

In the cohort examined, evidence during stage 1 of TDP-43 immunonegative nuclei in Betz cells that occurred in the absence of bulbar and spinal pTDP-43-immunopositive *α*-motoneurons was not found. Thus, it is impossible to know which of the two (neocortical or bulbar/spinal) sites becomes involved first. Nevertheless, it is remarkable that both of the sites that become involved very early, although spatially distant from one another, are directly connected by axonal contacts.

During subsequent sALS stages, additional nerve cell types are drawn into the pathological process: Parvocellular projection neurons of the red nucleus that receive cortico-rubral afferents become involved in stage 2 and consistently display pTDP-43 immunopositive cytoplasmatic inclusions [13]. Given that at stage 3 cytoplasmic aggregates also develop in the medium-sized striatal projection neurons that are controlled by corticostriatal projections [73], one is inclined to speculate that the rapid appearance in these subcortical target cells of cytoplasmic inclusions, which extend into the axonal compartment, might prevent pTDP-43 from being transferred to the next nerve cells in the neuronal chain where the pathological protein possibly could propagate new lesions. Is this the chief reason why neither the cells of the pallidum, which are contacted via striato-pallidal fibers, nor the Purkinje cells of the cerebellum, which ultimately are reached via the rubro-olivary and olivocerebellar pathway (climbing fibers), fail to reveal any signs of pathological involvement in sALS?

The question also arises of how much importance should be assigned to the monosynaptic contacts between the cortical Betz cells and bulbar/spinal *α*-motoneurons [12, 20, 44, 45, 56]. The cellular events that lead to the initial dysfunction of the normal nuclear protein TDP-43 in a given Betz cell are still not known, but the spread of the cortical pathology into additional neuronal types might take place directly (namely, monosynaptically) via transsynaptic transneuronal transmission to the subcortical neuron [12, 29, 42, 50, 69].

The existence of even the most subtle cytoplasmic inclusions in isolated Betz cells (Fig. 1i, n, o [arrows]; see also Fig. 3g) could mean that the protein does not completely forfeit its capacity to aggregate. On the other hand, the amount and proportions of these minor inclusions in relationship to the size of the voluminous cell nucleus appear to be disproportionate to the putative amount of intranuclear TDP-43 that was present originally (e.g., Fig. 1i). As such, it is conceivable that in sALS soluble but abnormally modified TDP-43 might be present in the cytoplasm in a state (for example, either highly diluted or with concealed epitopes) that does not produce a positive signal in anti-TDP-43 immunoreactions (indicated by the light pink-shaded cytoplasm in Fig. 3e). Of course, by way of comparison with the Betz cells, it also would be necessary to see if other neuronal types in the cerebral cortex, such as the pyramidal cells in the infragranular layers V and VI that give rise to the corticostriatal and the corticorubral tracts, display the same profile, namely, nuclear clearing in the absence of cytoplasmic inclusions. However, this is a technically difficult undertaking, inasmuch as these types of medium-sized pyramidal cells in layers V–VI lack distinctive morphological characteristics.

A further possible postulate would be that, it involved cortical Betz cells which display no or very subtle cytoplasmic inclusions, a soluble but abnormal TDP-43 protein develops that is pathogenic and, to the extent that it is aggregation-competent, also may be capable of seeding behavior analogous to the proteins tau and *α*-synuclein [21, 40]. This toxic TDP-43 protein may be transported through the axon and, via synaptic contacts [24], be transmitted from the primarily involved cortical Betz cells to hitherto uninvolved neurons, where it could then contribute to the renewed dysregulation of TDP-43 [15, 22, 35, 42, 43, 50, 62]. This line of thinking receives support from the combination of (1) the absence of pTDP-43 immunoreactivity in nerve cells located directly next to the primarily involved Betz cells in layer Vb (Fig. 2f, g) and (2) the conspicuous presence of pTDP-43 cytoplasmic inclusions that are confined only to distant but axonally interconnected *α*-motoneurons in the spinal cord and lower brainstem of the cases examined (Figs. 1e, 3f).

Human Betz cells establish direct synaptic (i.e., monosynaptic) contacts to bulbar and spinal *α*-motoneurons [39, 44–46, 63]. Bulbar and spinal *α*-motoneurons are controlled and contacted directly via the corticobulbar or corticospinal tracts [12, 20]. By contrast, the observation that the motoneurons for control of extrinsic eye muscles are mostly spared in sALS may be explained, in part, by the fact that these neurons are not directly under the control of corticofugal projections [13, 17, 58, 68]. The pre- and postsynaptic structures of the Betz cell monosynaptic contacts are presumably so well sealed off by astrocytic processes that the pathogenic molecules cannot diffuse from synaptic cleft into the interstitial space [26, 72]. With time, larger aggregates also develop in the cytoplasm of Betz cells (Fig. 1p), and it is conceivable that these lesions actually might reduce the danger emanating from the initially much smaller but still soluble and presumably toxic axoplasmic aggregates. That this postulated and incipiently soluble abnormal protein in the cytoplasm is not inconsequential becomes clear when one considers that involved Betz cells ultimately die prematurely, beginning in stage 1 (Table 2 D; Fig. 1q) [13].

The considerations above are related to the idea that sALS may be primarily a disorder of the human neocortex [12, 20]. This would make the neocortex virtually the only source of neuronal types that do not consistently produce somatodendritic and axonal aggregates out of the abnormally phosphorylated and misfolded TDP-43 in their cytoplasm. The neuronal inclusions that subsequently develop in bulbar and spinal *α*-motoneurons may be induced via corticofugal axons and via complete synapses that directly contact these neurons. These secondarily involved subcortical nerve cells could convert the pTDP-43 in their cytoplasm into insoluble inclusions and thereby prevent further propagation of the pathology. If these assumptions were to prove accurate and pending additional studies, it would be important to identify the cellular mechanisms that might cause TDP-43 in the cytoplasm of susceptible neocortical projection neurons to persevere there in a soluble state [28]. Of similar importance would be the identification of the pathways needed to induce the conversion of the potentially toxic soluble protein into inert and insoluble aggregates. A therapeutically induced conversion of soluble TDP-43 in descending neocortical axons into insoluble aggregates might hinder the pathological process and the further propagation or spreading of sALS [40, 42, 50, 59, 69].

In contrast to the hypothesis of anterograde degeneration discussed above, a ‘dying-back’ theory has been proposed, according to which the pathological process underlying sALS could originate either in spinal cord *α*-motoneurons [2, 18, 65], in the periphery at the neuromuscular junction [25], or in myocytes of the striated skeletal musculature [30]. This would mean that potentially toxic factors in spinal cord *α*-motoneurons would be taken up by or transferred to Betz cell axons and transported retrogradely to their somata, where physiological TDP-43 function could become perturbed without TDP-43 phosphorylation or with only minimal phosphorylation, thereby leading to a dysmetabolism of the Betz cells in the complete absence, or virtual absence, of somatic cytoplasmic aggregates. Convincing morphological evidence for a retrograde degenerative process does not currently exist. Although additional studies, including experimental models, are required to test both hypotheses [37], the changes in Betz cells reported here are more compatible with an anterograde pathological process.
